# PARP Inhibitors as a Therapeutic Agent for Homologous Recombination Deficiency in Breast Cancers

**DOI:** 10.3390/jcm8040435

**Published:** 2019-03-30

**Authors:** Man Yee T. Keung, Yanyuan Wu, Jaydutt V. Vadgama

**Affiliations:** 1 Division of Cancer Research and Training, Charles Drew University, Los Angeles, CA 90059-2518, USA; manyeekeung@cdrewu.edu (M.Y.T.K.); yanyuanwu@cdrewu.edu (Y.W.); 2 David Geffen School of Medicine, Jonsson Comprehensive Cancer Center, University of California at Los Angeles, Los Angeles, CA 90095, USA

**Keywords:** breast cancer, *BRCA*, BRCAness, PARP, PARP inhibitors, biomarkers, resistance

## Abstract

Poly (ADP-ribose) polymerases (PARPs) play an important role in various cellular processes, such as replication, recombination, chromatin remodeling, and DNA repair. Emphasizing PARP’s role in facilitating DNA repair, the PARP pathway has been a target for cancer researchers in developing compounds which selectively target cancer cells and increase sensitivity of cancer cells to other anticancer agents, but which also leave normal cells unaffected. Since certain tumors (*BRCA1/2* mutants) have deficient homologous recombination repair pathways, they depend on PARP-mediated base excision repair for survival. Thus, inhibition of PARP is a promising strategy to selectively kill cancer cells by inactivating complementary DNA repair pathways. Although PARP inhibitor therapy has predominantly targeted *BRCA*-mutated cancers, this review also highlights the growing conversation around PARP inhibitor treatment for non-*BRCA*-mutant tumors, those which exhibit BRCAness and homologous recombination deficiency. We provide an update on the field’s progress by considering PARP inhibitor mechanisms, predictive biomarkers, and clinical trials of PARP inhibitors in development. Bringing light to these findings would provide a basis for expanding the use of PARP inhibitors beyond *BRCA*-mutant breast tumors.

## 1. Introduction

Breast cancer is among the three most common cancers worldwide. It is also consistently the leading cause of disability-adjusted life-years (DALYs) and the leading cause of death in women [1]. Breast cancer comprises highly heterogeneous cancer sub-types, due to distinct genetic profiles manifesting in broad clinical presentations across patient populations. Breast cancers can be divided into molecular subtypes based on the presence of hormone receptors, estrogen receptor (ER) and progesterone receptor (PR), and receptors such as human epidermal growth factor receptor II (HER2) [2]. Triple-negative breast cancers, also called TNBC (ER negative, PR negative, and HER-2 negative), occur in approximately 17% of breast cancers, are aggressive cancers, and are associated with early development of metastasis [3]. Approximately 85% of all breast cancers are hormone receptor (HR) positive and can be treated with hormone therapies such as Tamoxifen, aromatase inhibitors, Trastuzumab (Herceptin), and others [3]. On the contrary, treatment of TNBC remains challenging, as this sub-type does not overexpress receptors for which hormone therapies can target. Additionally, HR-positive breast cancers, which initially responded well to hormone therapy, can develop into recurrent tumors with downregulation or loss of ER or PR, a finding which may contribute to resistance to endocrine therapy and highlights the challenges associated with breast cancer treatment [4,5,6].

*BRCA* mutation status is thus one phenomenon being studied for developing targeted therapies against TNBC and other breast cancer subtypes. Patients with TNBC have a higher prevalence of *BRCA1/2* mutations; however, prevalence varies from 10% to 42% [7]. Conversely, over 80% of breast cancer patients with a *BRCA1* mutation have TNBC [8]. *BRCA-*mutation associated tumors lack the homologous recombination repair (HRR) pathway, which repairs double-strand breaks (DSB). Published clinical studies suggest that germline *BRCA* mutation-associated (gBRCAm) breast cancers are more sensitive to DNA-damaging therapies like poly (adenosine diphosphate ribose) polymerase (PARP) inhibitors [9]. The FDA (Federal Drug Administration) has approved use of PARP inhibitors (PARPi) for treatment of ovarian, prostate, pancreatic, fallopian tube, and peritoneal cancers [10]. More recently in 2018, two PARPi, olaparib and talazoparib, were approved as monotherapies for *BRCA*-mutated HER2-negative metastatic breast cancer [11,12]. With the field of PARP inhibitor therapy rapidly advancing, PARPi warrant further clinical evaluation for treating the heterogeneous nature of breast cancer subtypes.

This review article explores the therapeutic potential of PARPi in cancer therapy and the use of PARPi beyond *BRCA*-mutant tumors, such as those with BRCAness and homologous recombination deficiency. An emphasis is placed on the components of the PARP pathway which mediate selective cytotoxicity of PARPi, predictive biomarkers, and clinical trials involving PARPi for the treatment of breast cancers. In this review, we also discuss the resistance mechanisms emerging against PARPi.

## 2. PARP Inhibitors as Therapeutic Intervention

PARP-1 and PARP-2 are the most extensively studied PARPs for their role in DNA damage repair. When induced by DNA lesions, PARP1 detects the DNA damage break and catalyzes the addition of poly (ADP-ribose) (PAR) chains to target proteins. This post-translational modification catalyzed by PARP, known as PARylation, mediates the recruitment of additional DNA repair factors to the DNA lesion [13,14]. Four known domains of PARP-1 facilitate DNA repair: (1) the amino-terminal DNA-binding domain containing two zinc-finger motifs, which recognize DNA strand breaks; (2) domain B, which contains a nuclear localization signal; (3) a central auto-modification domain; and (4) a carboxyl-terminal catalytic domain (CAT) that binds oxidized NAD^+^ [13,15,16,17,18]. A distinction is made that some researchers include the WGR (Trp, Gly, Arg) domain as part of CAT [16], while other researchers list the WGR and CAT as two separate entities (this distinction is further revisited in Section 2.1 of this paper) [19]. A 2012 review by Gibson and Kraus highlights the complexity of PAR and PARP interaction and the necessity of PARylation to disrupt protein–protein and protein–DNA interactions [20]. In addition, PAR is required for protein localization and formation of functional molecular scaffolds [20]. Another review from 2008 highlights research which examines the role of NAD^+^ concentration in compaction or decompaction of chromatin by PARP-1 [21]. In focusing on PARP’s role in DNA repair, upon PARP binding to a DNA strand break, PARylation is stimulated more than 500-fold [15,18]. In a complex mechanism involving recruitment of essential repair proteins, which include, but are not limited to, DNA ligase III, XRCC1, BARD1, γH2AX, 53BP1, *BRCA1,* and others, PARP-1 in conjunction with PARP-2 and PARP-3 facilitate repair via multiple repair processes mentioned below. The involvement of these PARPs in repair mechanisms have been reviewed extensively by Beck et al. [22].

Although our understanding of PARP is largely connected to HRR, PARP-1 also influences multiple repair pathways that can be utilized by BRCA1/2-deficient cells. While homologous recombination is the principle DSB repair mechanism, in homologous recombination-deficient cells, such as those with *BRCA1/2* mutations, DNA damage is either repaired with low fidelity repair mechanisms or is not repaired at all [19]. These alternative pathways include non-homologous end-joining (NHEJ), alternative non-homologous end-joining (aNHEJ), and single-strand annealing (SSA), pathways which are generally considered to be error-prone compared to HRR [23]. NHEJ also plays a large role in DSB repair; in contrast to HRR, NHEJ does not require a homologous template for DNA repair [24]. NHEJ can be categorized into two subtypes, classical NHEJ or aNHEJ, which have varying consequences for genome integrity. One potential reason for the perceived fallibility of classical NHEJ may be because the NHEJ machinery is flexible in dealing with a wide range of DSB structures [25]. Alternatively, aNHEJ is involved in repairing junctions with complex insertions/deletions, although research has shown that aNHEJ joins DSBs on different chromosomes, leading to chromosomal translocations and mutagenic consequences [26]. SSA also repairs DSBs, but extensive resection occurs. Following annealing at direct repeats flanking the DSB and 3’ flap removal, single-strand gap filling and ligation results in large deletions [27]. Although PARP and HRR are largely intertwined in the context of PARP inhibitors, the existence of alternative repair pathways may explain how cells can develop resistance to PARP inhibitors. 

**Figure 1:**
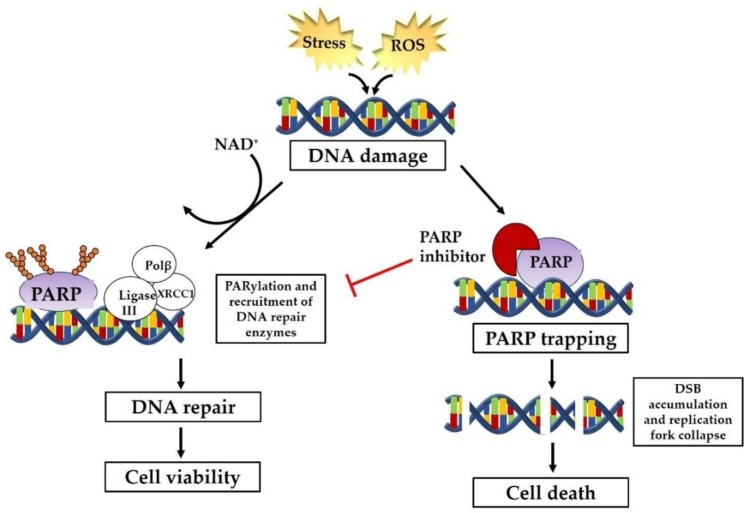
PARP pathway overview. Cellular stress such as oxidative stress from reactive oxygen species causes DNA damage in the form of single- and double-strand breaks. Under normal conditions, the PARP pathway is activated. ADP-ribose units are recruited to sites of DNA strand breaks in a process known as PARylation. With the assistance of PARP and other DNA repair enzymes, repair of DNA strand breaks occurs, and the cell remains viable. This figure provides an overview of what happens in the presence of a PARP inhibitor in BRCA-mutated cells which have defects in the homologous recombination repair pathway. The PARP inhibitor mediates inhibition of PARylation, thereby preventing repair of DNA strand breaks via the PARP pathway or the homologous recombination repair pathway. This synthetic lethality in which both repair pathways are nonfunctional contributes to unrepaired single-strand breaks and double-strand breaks; accumulation of double-strand breaks ultimately leads to apoptosis and cell death. (DSB = double-strand break; PARP = poly (ADP-ribose) polymerase; ROS = reactive oxygen species)

In the presence of DNA damage and a PARP inhibitor, PARP inhibitors prohibit PARylation from occurring and PARP-1 remains tightly bound to damage sites. This action in which the binding affinity of PARP-1 to damaged DNA is increased is called PARP trapping [28]. Murai et al. propose two mechanisms by which PARPi trap PARP, including the inhibition of PARylation or a reverse allosteric mechanism in which PARPi binds to NAD^+^ and enhances the binding of DNA to PARP’s zinc finger domain [28]. As a result of PARP trapping, the following occur: replication fork collapse, unrepaired DNA damage, and cytotoxicity (Figure 1) [29]. The synthetic lethality mediated by PARP inhibition and homologous recombination deficiency (HRD) leads to cell death. Early studies demonstrate that cells that are deficient in *BRCA1* or *BRCA2* display increased sensitivity to PARP inhibitors by about 1000-fold [9]. Thus, PARP inhibition is a promising therapeutic strategy for homologous recombination-deficient tumors, such as those associated with *BRCA* mutations, and the use of PARPi can potentially be expanded to tumors without *BRCA* mutations.

### 2.1. Selective Cytotoxicity of PARP Inhibitors 

In looking at how PARP inhibition may mediate selective cytotoxicity, it is essential to revisit the relative contributions of both catalytic domain function and PARP trapping. Firstly, previous research has emphasized the importance of particular PARP-1 domains (out of the domains mentioned in the previous section), which are essential for formation of interdomain contacts linking the DNA domain interface to CAT. These domains include Zn1 and Zn3 (part of the DNA-binding domain) and WGR which are linked to CAT [19]. As mentioned previously, there is discrepancy as to whether or not WGR is part of the CAT domain. A DNA-dependent mechanism, mediated by the automodification domain of PARP-1, involves binding of Zn1, Zn3, and WGR to a DNA strand, resulting in distortion of the hydrophobic core of the helical subdomain (HD) of CAT. HD distortion results in decreased thermal stability of CAT, thereby increasing CAT domain exposure. However, in the folded state, HD inhibits catalytic activation of PARP-1 [30]. More recent research further probes the interaction of CAT and NAD^+^. Results show that while PARP-1 alone has less access to NAD^+^, thus accounting for the minimal level of unstimulated PARP-1 activity, PARP-1 when bound to DNA strand breaks allows NAD^+^ to access CAT [31]. When the NAD^+^ binding site is occupied, distorted HD is stabilized and affinity for DNA strand break damage is increased [31]. Thus, the network of multi domain assembly is critical for DNA-dependent activation of full-length PARP-1. Altered domain interfaces are the sites at which PARP inhibitors can disrupt allosteric interaction, thereby rendering PARP-1 nonfunctional.

Secondly, selective cytotoxicity of PARPi may be mediated by PARP trapping, a term coined by Pommier and colleagues to refer to the effect of PARPi sequestering both PARP-1 and PARP-2 on PARP–DNA complexes, blocking the recruitment of additional DNA repair proteins [28]. This study is one of the first to examine the idea that PARPi treatment results in varying degrees of toxicity, based not on its potency to inhibit PARP catalytic activity but rather on its ability to stabilize PARP-DNA complexes. In the presence of both PARPi and damaged DNA, the distribution of PARP-1 shifts from nuclear-soluble to chromatin-bound [28]. A 2015 study by Hopkins et al. further expands upon the PARP trapping concept but instead supports that catalytic inhibition and PARP trapping are, in fact, positively correlated in biochemical systems but not in cells [32]. Furthermore, these studies reveal that although the developed PARP inhibitors (olaparib, niraparib, rucaparib, talazoparib, and veliparib) have shown effective inhibition of PARP-1 and -2, they differ in their ability to cause PARP trapping, with talazoparib having the highest trapping activity [32,33,34]. Accordingly, it appears that the cytotoxic mechanism as it relates to PARP trapping may be dependent on the use of PARPi in monotherapy or combination therapy. For example, when PARPi is combined with topoisomerase I (TOP1) inhibitors, cytotoxicity appears to be less attributed to PARP-1 trapping [35]. On the other hand, the activity of PARPi plus alkylating agents, such as temozolomide, appears to be largely attributed to PARP trapping, as demonstrated by quantification of PARP–DNA complexes following treatment [36].

Recent research elaborates upon the cellular consequences of PARP trapping and establishes the connection between ribonuclease (RNase) H2 deficiency and hypersensitivity to PARPi [37]. RNase H2 is involved in ribonucleotide excision repair. The evidence gathered by Zimmerman et al. shows that PARP inhibition requires the formation of trapped PARP1-DNA adducts in order to be synthetic lethal, as confirmed by double mutant RNASEH2A^KO^PARP1^KO^ cells. Their data further supports that ribonucleotides may be a major source of PARP trapping. They propose that both the canonical ribonucleotide excision repair (RER) pathway and TOP1-mediated ribonucleotide cleavage are involved in trapping of DNA lesions that impede DNA replication and ultimately lead to cell death.

These mechanistic findings have further implications for the use of PARPi in combination with other anti-cancer agents and when considering toxicity in clinical applications. 

## 3. BRCAness and Homologous Recombination Deficiency (HRD)

Accordingly, the connection between TNBC and *BRCA* mutations has been widely studied and utilized as a prognostic marker for TNBC therapy. *BRCA1* tumors are often grade 3 ductal carcinomas and ER−, while *BRCA2* tumors are often ER+ and lower grade with high mitotic rates [38,39]. However, another aspect complicating TNBC therapy is BRCAness, which defines molecular and phenotypic characteristics that TNBC sporadic cancers share in common with *BRCA*-mutated cancers [8]. 

Tumors which exhibit BRCAness may also exhibit loss-of-function alterations causing a dysfunction in the HRR pathway, even though they do not have a mutation in the *BRCA* gene [40]. The application of mutational signatures, which are patterns of somatic mutations that reflect levels of DNA damage and repair processes, to the understanding of BRCAness is increasingly studied [41,42]. Numerous studies have revealed mutational signatures associated with breast cancer, namely signatures 1, 2, 3, 8, and 13, and also provide a mutational characterization of cancers with *BRCA1* and *BRCA2* [43,44]. Polak et al. explores signature 3, a mutational signature prevalent in tumors with BRCAness [45], and finds altered expression of *PALB2* and *RAD51,* genes important for the HRR pathway. Additionally, signature 3 was found in tumors with both germline and somatic *BRCA1* and *BRCA2* mutations [45]. Thus, the role of signature 3 as a potential biomarker (more on mutational signatures in Section 4.1) needs to be investigated further, but signature 3 can help guide development of BRCAness-targeting therapies.

Identifying HRD in tumors is complex and involves detecting mutational alterations in the genome. Alterations can come in the form of germline mutations in HRR-associated genes, such as *PALB2*, *BARD1*, and *RAD51D* [46]. Somatic mutations may also arise in HRR-associated genes; however, germline mutations are more common than somatic mutations in *BRCA1-* and *BRCA2-* mutated cancers [43]. BRCAness and HRD also involves methylation of the *BRCA* promoter, which appears to be most common in TNBCs [8,40,47]. Breast tumors with *BRCA1* methylation also show higher histological grade, like that of *BRCA1*-mutated tumors [8]. It was demonstrated that patients with *BRCA*-mutated tumors had a significantly higher pathological complete response (pCR) to chemotherapy (63%), while tumors with BRCAness only had a pathological complete remission rate of 35% [8].

**Figure 2:**
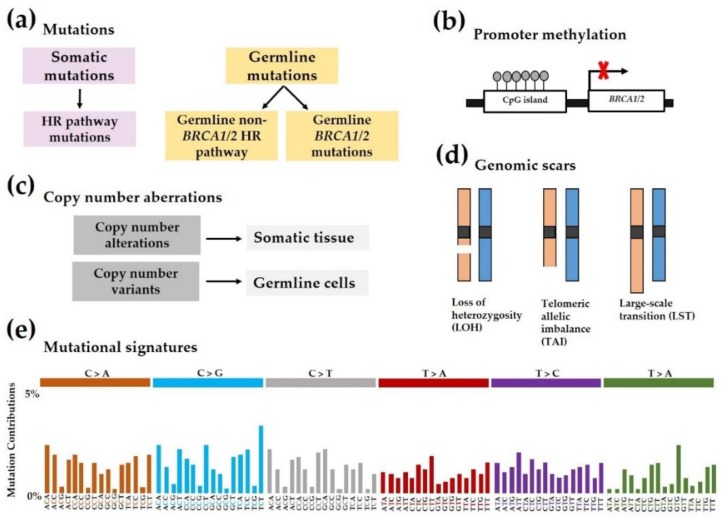
Homologous recombination deficiency (HRD) and genomic alterations. (**a**) Both germline mutations and somatic mutations involving HR-related genes, including *BRCA1* and *BRCA2*, may be associated with HRD. (**b**) Promoter methylation involves addition of a methyl group to CpG islands, which ultimately silences gene expression. This is an epigenetic mechanism implicated in HRD. (**c**) Copy number aberrations/alterations alter chromosomal structure and are a hallmark of HRD. (**d**) Genomic scars are examined and can be scored to measure the level of HRD phenotype in a sample. (**e**) Mutational signatures are patterns of base pair mutations that measure levels of DNA damage in a sample. Pictured here is a representation of signature 3, a mutational signature highly prevalent in tumors with BRCAness and also one of a few distinct mutational signatures found in breast cancer. Signature 3, among other mutational signatures, was characterized in 2013 by Alexandrov et al. [42].

Additional contributors to HRD involve copy number alterations (CNA), and early research has revealed that the basal-like subtype of breast cancer has higher numbers of gains/losses, while the luminal B subtype has more frequent high-level DNA amplification [48]. DNA microarrays and comparative genomic hybridization (CGH) can be used to identify genome instability comparing test DNA and control DNA [49]. Chromosomal rearrangements, such as inversions, translocations, and recombination, are largely implicated in loss of heterozygosity (LOH) and allelic imbalance, which can be studied using single-nucleotide polymorphism (SNP) analysis [50]. Figure 2 summarizes the major biomarkers of HRD.

Increasingly studied is the involvement of microRNAs (miRNAs) targeting DNA repair machinery as a potential therapeutic target for BRCAness. Overexpression of miR-182 sensitizes MDA-MB-231 TNBC cells to PARPi by downregulating *BRCA1* [51]. Another study shows that miR-151-5p is upregulated in PARP1-overexpressing BRCA-mutated and sporadic breast tumors, which eventually could be considered as BRCAness tumors. This miRNA targets the coding sequence of *SMARCA5*, an ATP-dependent chromatin remodeler [52]. Additionally, numerous studies have explored the concept of BRCAness induction followed by treatment with PARPi. TGFβ induces BRCAness by downregulating *BRCA1*, *ATM*, and *MSH2*, genes involved in DNA repair, through a miRNA-mediated mechanism [53]. Another approach that has been explored is inducing BRCAness with histone deacetylase inhibitors [54,55].

## 4. Predictive Biomarkers in Breast Cancer

As PARPi selectively target HR-deficient tumors, particularly in patients who bear germline *BRCA1* or *BRCA2* mutations, reliable biomarkers are needed to accurately select patients with HR deficiency who would greatly benefit from PARPi-based anticancer therapies. There currently is no set method to determine whether a patient will respond to PARPi. However, in vitro and in vivo studies also demonstrate that HRD is not the only factor which determines sensitivity to PARPi. Thus, various biomarkers beyond tumor DNA repair status have also been explored.

One well-studied phenomenon of HRR is RAD51 localization to the nucleus after damage to DNA occurs. RAD 51 nuclear foci do not form in cells that lack functional *BRCA1/2* or other functional HRR components [56]. In establishing a connection between patient breast tumor samples and RAD51 formation, breast tumor samples were irradiated and subjected to RAD51 ionizing radiation induced foci (IRIF) analysis [57]. Over 10% of tumors showed impaired RAD51 IRIF formation from hypermethylation of the *BRCA1* promoter, which was also correlated with *BRCA* gene status and TNBC. RAD51 IRIF-negative tumors were sensitive to olaparib treatment. One major downside of the RAD51 IRIF assay is that it can only be performed on fresh tumor material. A more recent study establishes a connection between the presence of RAD51 nuclear foci formation and PARPi resistance in gBRCAm breast and ovarian tumors [58]. There are two major limitations to using RAD51 foci as a biomarker for HRR proficiency: (1) RAD51 foci formation must be induced by DNA damage, such as damage cause by PARPi; and (2) RAD51 is only detected in the S and G2 phases of the cell cycle [59]. 

Recently, genomic analysis revealed that two BC cell lines exhibit BRCAness, HCC70 (TNBC, basal, *BRCA* wt) and MDA-MB-468 (TNBC, basal, *BRCA* mutant) [60]. The other cell lines in this study, MCF7 (luminal A), MDA-MB-231 (TNBC, mesenchymal, *BRCA* wt (Wildtype)), and MDA-MB-436 (TNBC, mesenchymal, *BRCA* mutant), did not show a BRCAness phenotype but were categorized under sporadic-like breast tumors. In BRCA1-like cell lines compared with *BRCA1* wt cell lines, nine metabolites were identified to be significantly under-represented [60]. 

### 4.1. Sequencing-Based Methods

HRD scores are capable of detecting HRR defects and are reported to be predictive of response to PARPi in patients with TNBC and *BRCA1/2*-mutated breast or ovarian cancers [47,61]. HRD score has previously been shown to be predictive of tumor response to neoadjuvant chemotherapy [62,63]. Myriad Genetics, Inc. and Timms et al. developed a next-generation sequencing-based HRD assay using SNP profiling. This combines three different DNA-based metrics of genomic instability: HRD-loss of heterozygosity score (HRD-LOH) [61], HRD-telomeric allelic imbalance score (HRD-TAI) [64], and HRD-large-scale state transition score (HRD-LST) [65]. The HRD scores in their 2014 study examined breast tumors representing TNBC and all *ER/HER2* subtypes, and showed a significant association with *BRCA1/2* deficiency regardless of subtype or stage [47]. The presence of high HRD scores in *BRCA1/2*-intact tumors also strongly suggests that alternate mechanisms account for HRD [47].

Unique mutational signatures result from the use of error-prone DSB repair mechanisms in HRD tumors. In a 2013 study by Alexandrov et al., 20 distinct, conserved mutation signatures were present across multiple cancer types [42]. These signatures are characterized by the presence of small deletions of up to 50 bp. In breast cancer specifically, signatures 1B, 2, 3, 8, and 13 were prevalent and strongly associated with *BRCA1/2* mutations. These signatures may be examined to identify tumors with a BRCAness phenotype and presumably predict response to PARPi therapy [50], although genomic scars in gBRCAm tumors may persist after restoration of HRR function [50,66]. Other gene-specific sequencing strategies include multiplex-ligation-dependent probe amplification (MLPA) [67] and identification of transcriptional metagene signatures [68].

HRDetect is a whole genome sequencing-based predictor that was developed to accurately detect *BRCA1/2*-deficient breast tumor samples [66]. In a 2017 paper describing HRDetect, six predictive parameters were observed in distinguishing BRCA1- and BRCA2-deficient tumors, in order of decreasing weight: microhomology-mediated deletions, base-substitution signature 3, rearrangement signature 5, rearrangement signature 5, HRD index, and base substitution signature 8 [66]. This final predictor, termed HRDetect, was applied to a cohort of 560 cases of breast cancer and showed a 98.7% of detecting *BRCA1/2-*deficient tumors. This method analyzes multiple mutational signatures as a predictor, which is advantageous, the authors say, to using the HRD score. Additionally, HRDetect is able to distinguish *BRCA1*-deficient from *BRCA2*-deficient tumors. 

Numerous diagnostic tests have been explored to predict sensitivity to PARPi. Myriad Genetics, Inc. has also developed BRACAnalysis CDx, the first FDA approved companion diagnostic for treatment in patients with gBRCAm ovarian cancer and metastatic breast cancer. BRACAnalysis CDx is a diagnostic test which utilizes sequencing and deletion/duplication analysis to identify germline mutated *BRCA1/*2 genes [69]. The effectiveness of BRACAnalysis CDx was established based on the OlympiAD trial population (NCT02000622), leading to the FDA approval of olaparib for ovarian cancer patients with gBRCAm in 2014 [69,70]. Foundation Medicine’s T5 NGS assay (Cambridge, MA, USA) was used to assess genomic LOH when predicting response of patients with recurrent ovarian carcinoma to rucaparib [71]. Other diagnostic tests include Foundation Medicine’s FoundationOne CDx, the first comprehensive genomic profiling test for all solid tumors, and MSK-Impact, which is capable of detecting protein-coding mutations, copy number alterations, and selected promoter mutations and structural rearrangements in 341 cancer-associated genes [72,73]. The main takeaway is that, along with the identification of predictive biomarkers, whole-genome sequencing and sequencing-based diagnostic or predictive methods can support the implementation of using PARPi for precision medicine. However, these studies also reveal that sensitivity to PARPi is not limited to those with *BRCA1/2* mutations. The challenge of finding predictive biomarkers for response to PARPi underscores the urgent need to develop targeted therapies to approach the heterogeneous nature of breast cancer.

## 5. PARP Inhibitors as a Single Agent

In 2005, through animal model experiments, Bryant et al. and Farmer et al. confirmed the use of PARP inhibitors as a target therapy in *BRCA*-mutated cells [9,74]. Subsequently, following strong preclinical findings demonstrating the therapeutic potential of PARP inhibitors, olaparib showed durable antitumor responses in patients with advanced gBRCAm ovarian, breast, or castration-resistant prostate cancer [75,76]. Olaparib was the first PARPi approved by the FDA in December 2014 for use as monotherapy in patients with gBRCAm advanced ovarian cancer. Since then, the field of PARPi monotherapy has rapidly advanced, with additional FDA approvals of niraparib (March 2017) and rucaparib (April 2018) for maintenance treatment of BRCA-mutated ovarian cancer. In 2018, two PARP inhibitors were approved for gBRCAm metastatic breast cancer, olaparib and talazoparib.

## 6. Combination Strategies with PARP Inhibitors

Previous in vitro studies have examined various mechanisms to increase tumor sensitivity to and to maximize the efficacy of PARPi through combination therapy. A critical concern is the development of resistance to PARPi therapy (please refer to Section 8, “Acquired resistance to PARP inhibitors”), which also can be mediated through the use of PARPi in combination with other agents. Additional concerns include increased cytotoxicity or adverse events with combination therapy, which has been addressed in other reviews.

The effect of PARPi on cancer cells is enhanced in combination with DNA demethylating agents [77]. This study showed that PARP-1 and DNA methyltransferase 1 (DNMT1) form a complex and are recruited to sites of DNA damage. Combination drug administration yielded increased cytotoxicity and frequency of DSBs compared to either PARPi or DNMTi alone. In vivo, TNBC xenografts treated with a DNMTi–PARPi combination demonstrated a significant reduction in tumor burden in comparison to controls or monotherapy [77].

A 2012 study examines the efficacy of PARP inhibitors in patient populations other than those with *BRCA*-mutant tumors. This study focuses on *BRCA*-proficient TNBC and sensitizes these cells to PARP inhibition via inhibition of the phosphoinositide 3-kinase (PI3K) pathway [78]. Under normal conditions, PI3K stabilizes DNA repair by interacting with the HRR complex [79]. Treatment of *BRCA* wt TNBC cells with BKM120, a pan-PI3K inhibitor, resulted in downregulation of *BRCA1/2* along with an increase in PAR proteins. Combination of olaparib, a PARPi, with BKM120 showed a significant reduction in *BRCA* wt TNBC cells. In patient-derived primary tumor xenografts, the TNBC models studied, which demonstrated downregulated *BRCA* expression upon BKM120 treatments, also showed an increase in PAR levels. While olaparib monotherapy was ineffective in these xenograft models, BKM120 and olaparib combination yielded significantly decreased tumor growth. Of note, BRCA downregulation and PARP activation was required for the dual effect of PI3K inhibition and PARP inhibition.

Many additional pathways have been studied to increase sensitivity of tumor cells to PARP inhibitors. As mentioned previously, breast cancer heterogeneity is challenging when it comes to treatment and prevention. Understanding the mechanistic pathways whereby PARP is affected is crucial to effectively target both *BRCA* wt and *BRCA*-mutant breast tumors.

## 7. Clinical Development of PARP Inhibitors in Breast Cancer

The following discussion examines developed PARP inhibitors that have shown effective and comparable inhibition of PARP-1 and PARP-2. However, these PARP inhibitors differ in PARP-trapping activity. Discussions of PARPi are organized in order of increasing PARP-trapping activity, from veliparib with the least PARP-trapping activity to talazoparib with the most PARP-trapping activity, and rucaparib, niraparib, and olaparib in between [28,32,33,34].

There are over 150 completed, recruiting, or current registered clinical trials (clinicaltrials.gov) involving the PARP inhibitors niraparib, olaparib, rucaparib, talazoparib, and veliparib. A majority of these clinical trials involve PARPi as monotherapy in patients with BRCA-mutated tumors, especially with those involving breast cancer. Several clinical studies in ovarian cancer; however, highlight the utility of PARPi to treat non-BRCA-mutated tumors: ARIEL2 (NCT01891344) [80], ARIEL3 (NCT01968213) [71], NOVA (NCT01847274) [81], Study 19 (NCT00753545) [81]. 

Combination trials include PARPi with platinums, taxanes, ATM (Ataxia Telangiectasia Mutated Protein) inhibitors, ATR(Ataxia Telangiectasia and Rad3-related Protein) inhibitors, Wee1 inhibitors, and PI3K inhibitors. Combination immune therapy is explored as well. Thus far, olaparib, niraparib, and rucaparib have been FDA approved for use as maintenance therapy in recurrent ovarian cancer. Only olaparib and talazoparib have been approved by the FDA for treatment of gBRCAm breast cancer.

### 7.1. Veliparib (ABT-888)—AbbVie

#### 7.1.1. Monotherapy

A phase I trial completed in 2014 showed evidence of veliparib’s anti-tumor activity for patients with gBRCAm advanced TNBC and ovarian cancers (NCT00892736) [82]. In this study, objective response rate (ORR) was 23%, and (CBR) was 58%. The suggested maximum tolerated dose was 400 mg twice daily. Twenty-eight patients with gBRCAm had an ORR of 40% and clinical benefit rate (CBR) of 68%. However, with limited anti-tumor effect of veliparib monotherapy, combination therapies involving veliparib were initiated.

#### 7.1.2. Combination Strategies

Veliparib has been widely studied in combination with several other regimens. Early phase I and phase II trials studied veliparib in combination with carboplatin and paclitaxel (NCT00535119) [83], veliparib in combination with doxorubicin and cyclophosphamide (NCT00740805) [84], veliparib with cisplatin and vinorelbine (NCT01104259) [85], veliparib with carboplatin (NCT01251874) [86], and veliparib with temozolomide (NCT01009788) [87]. In these early combination studies, a 73% response rate was seen with veliparib in combination with cisplatin and vinorelbine in BRCA-mutated TNBC [85], and modest response rates with the other aforementioned combinations, but adverse events included neutropenia and thrombocytopenia [83,84,85,86,87]. 

BrighTNess is a phase III randomized, double-blind, placebo-controlled trial involving patients with stage II–III TNBC treated with paclitaxel and/or veliparib with or without carboplatin (NCT02032277) [88]. Patients who were treated with the triple combination therapy of paclitaxel, carboplatin, and veliparib achieved pCR of 53% (168/316), compared to 31% (49/158) in patients who received paclitaxel alone. Veliparib did not substantially increase toxicity, compared to patients who had received carboplatin and experienced serious adverse events such as neutropenia, anemia, and thrombocytopenia. Results of triple combination therapy were similar to results of treatment with paclitaxel and carboplatin (58%, 92/160). 

BROCADE 3 (NCT02163694) is a placebo-controlled, randomized phase III trial that is currently open for patients with gBRCAm HER2-negative advanced or metastatic breast cancer. Patients receive carboplatin and paclitaxel with or without veliparib. The estimated study completion date is May 2019.

### 7.2. Niraparib (MK4827)—Tesaro

Niraparib is a potent oral inhibitor of PARP-1 and PARP-2 with IC_50_ values of 3.8 and 2.1 nmol/L, respectively [89,90]. Niraparib is approved by the FDA for maintenance treatment of patients with recurrent high-grade serous epithelial ovarian, fallopian tube, or primary peritoneal cancer with complete or partial response to platinum-based chemotherapy [91].

#### 7.2.1. Monotherapy

In a phase I dose escalation study (NCT00749502) [92], four of 12 breast cancer patients were *BRCA1/2*-mutant carriers. Two (50%) of the four *BRCA* mutant carriers had 30% and 50% partial response rate (PRR) to niraparib, respectively. PR lasted approximately three months at starting doses of 210 and 150 mg/day. The most common adverse effect to niraparib therapy was anemia, nausea, fatigue, and thrombocytopenia. The recommended phase 2 dose was 300 mg PO (per os, by mouth) daily. Although a small sample size is noted, this study demonstrated that niraparib had durable antitumor activity in *BRCA-1/2* mutant carriers in patients with breast cancer [92]. The BRAVO (A Phase III Trial of Niraparib Versus Physician’s Choice in HER2 Negative, Germline BRCA Mutation-positive Breast Cancer Patients) study was initiated in late 2013 (NCT01905592). This randomized, open-label, multicenter, controlled study is a comparison of progression-free survival (PFS) treated with niraparib versus physician’s choice of chemotherapy (PCT) (eribulin, vinorelbine, gemcitabine, or capecitabine). Niraparib is administered once daily during a 21-day cycle. Physician’s choice is administered on a 21-day cycle. A phase II study (NCT02826512) initiated in 2018 is planned to assess the feasibility of niraparib in BRCA1-like, HER2-negative breast cancer patients.

#### 7.2.2. Combination Strategies

A phase I/II study named TOPACIO (NCT02657889), initiated in March 2016, examines niraparib used in combination with an anti-PD-1 monoclonal antibody, pembrolizumab (MK-3475), to treat patients with advanced breast cancer, TNBC, or ovarian cancer. Phase I of the study involves administering ascending doses of niraparib up to 300 mg/day orally on day one to 21 of a 21-day cycle, with IV pembrolizumab 200 mg on day one of each 21-day cycle. In phase II, patients are administered niraparib in combination with IV pembrolizumab 200 mg on day one of each 21-day cycle. This study’s projected completion date is February 2019.

### 7.3. Olaparib (AZD2281, Ku-0059436, Lynparza)—AstraZeneca

#### 7.3.1. Monotherapy

Olaparib was the first PARP inhibitor to be approved for cancer therapy. Phase I results in 2009 from Fong et al. demonstrated that olaparib had antitumor activity in patients with gBRCAm ovarian and breast cancers and advanced solid tumors (NCT00516373) [76]. A subsequent phase II trial (ICEBERG, NCT00494234) by Tutt et al. in patients with confirmed BRCA mutations showed a favorable therapeutic index, providing proof of concept for the efficacy of olaparib, with 41% (11/27) ORR in patients taking olaparib 400 mg BID [93]. Another early phase 2 study by Gelmon et al. (NCT00679783) demonstrated that patients with TNBC had no OR, while those with ovarian cancer had 41% OR [94]. A 2014 multicenter, open-label, noncomparative phase II clinical trial examined the efficacy of olaparib monotherapy in patients with advanced, recurrent cancer with a germline BRCA1/2 mutation. A total of 62 breast cancer patients were treated, and 60% of these breast cancer patients had a BRCA1 mutation. A total of 12.9% of the breast cancer patients had a tumor response rate, and 46.8% of patients achieved stable disease (SD) after ≥8 weeks olaparib treatment. Median duration of response was 204 days. A total of 29% of patients were progression free at six months. Median overall survival was 11 months, and 44.7% of patients were alive at 12 months. Anemia was the most common ≥grade 3 adverse event [95].

#### 7.3.2 Combination Strategies

In addition to numerous monotherapy studies, many studies now look at the response of olaparib in combination with other cytotoxic drugs (Table 1). A 2014 phase I study showed that BRCA1/2 breast cancer patients had a 71% response rate (12/17 patients) to olaparib (50 mg BID) and cisplatin (60 mg/m^2^) combination therapy. Frequent grade 3 toxicities, such as neutropenia, anemia, and leucopenia, rendered higher doses of olaparib (100 or 200 mg BID) and cisplatin (75 mg/m^2^) intolerable [96]. 

In another recent phase I/Ib clinical trial, women with sporadic TNBC were treated with a combination of olaparib and carboplatin. Twenty-eight patients were treated on a 21-day cycle, with 400 mg olaparib twice daily on days one to seven and carboplatin on day one or two. The most frequent non-hematologic events included nausea, fatigue, headache, gastroesophageal reflux disease, and skin rashes. Hematologic toxicity was very common, with thrombocytopenia being the most frequent occurrence in 50% of patients. The patients in this study were heavily pretreated; 75% of the patients had been treated with over three prior therapies. Twelve of 28 patients achieved stable disease, but nine of the patients had progressive disease. One patient who was an exceptional responder had CR for 69+ months [97].

Olaparib also demonstrated anti-tumor activity when used in combination with rapamycin [98]. Another combination phase I study with olaparib and BKM120, a PI3-kinase inhibitor, demonstrated clinical benefit for patients with high grade serious ovarian cancer or TNBC [99]. A phase 1 study examining olaparib and cediranib (angiogenesis inhibitor) in ovarian cancer and TNBC determined the recommended phase 2 dose of cediranib to be 30 mg daily and olaparib 200 mg BID [100]. Although ORR in ovarian cancer patients was 44%, none of the breast cancer patients in this study achieved clinical response. 

Approval of olaparib for gBRCAm HER-2-negative breast cancer came following results from the phase III OlympiAD clinical trial funded by AstraZeneca (NCT02000622) [70]. This was a randomized, controlled, open-label, multicenter, international phase 3 trial. Patients received olaparib tablets (300 mg twice daily), and 97 patients received standard therapy which consisted of a single-agent chemotherapy (capecitabine, eribulin, or vinorelbine) according to the healthcare provider’s recommendation. In the olaparib group, median PFS survival was ~67% longer than the standard-therapy group (7.0 months with olaparib vs. 4.2 months with standard therapy). The response rate was 59.9% in the olaparib group (100 patients out of 167) and 28.8% in the standard-therapy group (19 of the 66 patients). Adverse events rated grade 3 or higher occurred in 36.6% of patients in the olaparib group and 50.5% in the standard-therapy group. 

Olaparib was approved by the FDA in January 2018 for treatment of gBRCAm, HER2-negative metastatic breast cancers.

**Table 1:** Completed clinical trials with olaparib combination in breast cancer patients. (AUC = area under the curve; BID = bis in die, twice a day; CBR = clinical benefit rate; CR = complete response; ORR = objective response rate; PO = per os, by mouth; PR = partial response; QD = quaque die, once a day; RP2D = recommended phase 2 dose; SD = stable disease; TNBC = triple negative breast cancer).

Therapeutic Strategy; Phase	Patient Population; Number of Breast Patients	RP2D	Results	Grade 3–5 Adverse Events	Identifier; References
Olaparib with carboplatin; I	*BRCA1/2* mutation, sporadic TNBC; 8	Olaparib 400 mg PO BID days 1–7, carboplatin AUC5	CR, 23 months (2.4%, 1/8); PR, 10 months (75%, 6/8); SD, 14 months (12.5%, 1/8)	Neutropenia, thrombo-cytopenia, anemia	NCT01445418; [101]
Olaparib with carboplatin; I	TNBC; 10 (4 *BRCA*-mutant)	Olaparib 200 mg PO BID for 7 days; carboplatin AUC4 q21d	CR, 32 months (10%, 1/10); PR, ~9 months (30%, 3/10)	Neutropenia, anemia	NCT01237067; [102]
Olaparib with cisplatin; I	Metastatic, *BRCA*-mutant	Intermittent olaparib 50 mg PO BID days 1–5; cisplatin 60 mg/m^2^	ORR (71%, 12/19)	Neutropenia, anemia, lipase elevation	NCT00782574; [96]
Olaparib with paclitaxel; I	Metastatic TNBC; 19	Olaparib 200 mg PO BID; weekly paclitaxel, 3 weeks of 4-week cycle	PR (37%, 7/19); SD ≥ weeks (32%, 6/19)	Neutropenia	NCT00707707; [103]
Olaparib with cediranib; I	Recurrent TNBC; 8	Olaparib 200 mg PO BID; Cediranib 30 mg PO QD	No CR or PR; SD > 24 weeks (25%, 2/8)	Hypertension, fatigue	NCT01116648; [100]

### 7.4. Rucaparib (AG-014699, PF-01367338)—Clovis Oncology

#### 7.4.1. Monotherapy

Studies with rucaparib has predominantly involved ovarian cancer. However, there are multiple clinical studies underway for breast cancer patients. RUBY is a single arm, open-label, multicenter phase II trial currently recruiting patients with a BRCAness phenotype, as characterized by high genomic LOH and/or a somatic *BRCA* mutation, including *BRCA* methylation or other epigenetic mechanisms (NCT02505048) [104]. Of note, this trial excludes patients with a known *BRCA1/2* germline mutation. Patients will receive rucaparib 600 mg BID in 21-day cycles. 

A recently closed phase II trial in patients with BRCA mutations and advanced and metastatic breast cancer is a dose escalation trial with results pending (NCT00664781).

#### 7.4.2. Combination Strategies

Following results from an in vitro study demonstrating that rucaparib induces cytotoxicity in *BRCA1/2*-mutated cells [105], Drew et al. also investigated the effect of different dosing schedules of IV and oral rucaparib in patients with advanced breast and ovarian cancer with gBRCAm. This phase II, open-label, multicenter trial revealed that rucaparib was well-tolerated up to doses of 480 mg per day, but no dose-limiting toxicities (DLT) were seen in the IV phase. Of 78 total patients, 15 had *BRCA1* mutation-associated breast cancer and 12 had *BRCA2* mutation-associated breast cancer. Although there were no responders by tumor ORR in the breast cancer patients, 39% of the 23 breast cancer patients did achieve stable disease (SD) at ≥12 weeks [105]. The results of this study were more promising for the ovarian cancer patients, this study also demonstrated that continuous dosing orally QID or BID is more effective at maintaining PARP inhibition, compared to an intermittent dosing schedule [105,106]. The patients in this study achieved SD with the continuous dosing schedule. 

A phase I study in patients with advanced solid tumors combined rucaparib with carboplatin (NCT01009190) [107]. Twenty-two of the 85 patients enrolled had breast cancer, and one breast cancer patient achieved CR. Across all cohorts, the disease control rate was 68.8%. The maximum tolerated dose for the study was 240 mg per day when in combination with carboplatin. 

An ongoing phase II trial investigates the effect of rucaparib and carboplatin in patients with BRCA mutations and TNBC (NCT01074970). 

### 7.5. Talazoparib (BMN-673)—Pfizer, BioMarin, Medivation

Talazoparib has been demonstrated to have a high degree of potency and oral bioavailability, compared to the previously mentioned PARPi. In vitro studies from Shen et al. [108] show that talazoparib selectively targeted tumor cells with BRCA1/2 mutations from 20- to 200-fold greater potency than other PARP inhibitors. Significant antitumor effects were shown in vivo, and rats showed greater than 40% oral bioavailability. Following these promising results, multiple clinical trials with talazoparib monotherapy have been completed.

#### Monotherapy

A 2014 phase I trial examining antitumor activity of talazoparib (TALA) in gBRCAm breast cancer and metastatic small-cell lung cancer patients showed a 39% (7/18) partial response rate and a 6% (1/18) CR rate. Median PFS was 31 weeks for the breast cancer patients. The drug was administered on day one and eight to 35, followed by daily dosing in four-week cycles. Dosing was 1.0 mg/day [109].

A phase I dose-escalation trial (NCT01286987), completed in 2017, involved administering TALA either at nine dose levels, ranging from 0.025 to 1.1 mg/day (part 1), or at 1.0 mg/day (part 2) [110]. Of the 18 patients in part 1, those with a *BRCA2* mutation had higher ORR (55%), compared to those with a *BRCA1* mutation (38%). Higher clinical benefit rate (CBR) was seen in patients with a *BRCA2* versus *BRCA1* mutation (91% versus 50%, respectively). Of the 14 breast cancer patients participating in part 2, the ORR was 50% including one patient with a CR. Five of these patients had SD for 24 weeks, with a CBR of 86% lasting at least 24 weeks. Median PFS was 34.6 weeks. Additionally, although all patients had deleterious *BRCA1/2* mutations, patients with non-TNBC showed higher CBR (89% vs. 56%) and PFS (38.3 weeks vs. 20.4 weeks). Results from this phase I trial demonstrate the potency and tolerability of talazoparib, paving the way for the phase II ABRAZO trial and phase III EMBRACA trial. 

Patients with gBRCAm and advanced or metastatic breast cancer who had previously been exposed to platinum or multiple prior cytotoxic regimens were enrolled in the phase 2 ABRAZO trial (NCT02034916) [111]. Patients were divided into two cohorts: cohort 1 includes patients who have had platinum-based therapy, and cohort 2 includes patients who have had ≥3 platinum-free cytotoxic-based regimens. ORR was 21% in cohort 1 and 37% in cohort 2. Median PFS was 4.0 months and 5.6 months for cohort 1 and cohort 2, respectively. Common adverse events included anemia, fatigue, nausea, and diarrhea [111].

Prior to the EMBRACA clinical trial, Litton et al. conducted a feasibility study of administering TALA before neoadjuvant chemotherapy for breast cancer patients with a deleterious BRCA mutation [112]. These 13 patients had untreated, operable early-stage breast cancer. Median decrease in tumor volume was 88%. Adverse events included fatigue, dizziness, nausea, neutropenia, anemia, and thrombocytopenia. There were no grade 4 toxicities within the two months of talazoparib treatment. Consequently, the study was expanded to six months of TALA prior to surgery. In addition, patients were able to receive third-generation chemotherapy regimens after treatment. 

The open-label, randomized, phase III EMBRACA trial compares TALA to physician’s choice of therapy (PCT) in breast cancer patients with advanced disease and gBRCAm (NCT01945775) [113]. EMBRACA is the largest randomized trial evaluating a PARP inhibitor inpatients with gBRCAm and advanced breast cancer. 288 patients were treated with TALA and 144 were treated with PCT. Patients treated with TALA showed median PFS of 8.6 months compared to those treated with PCT of 5.6 months. Overall survival was 22.3 months in the TALA group and 19.5 months in the PCT group. ORR for TALA was also statistically significant compared to PCT, at 62.6% versus 27.2, respectively. Although grade 3–4 hematologic adverse events occurred more often in patients treated with TALA than PCT (55% vs. 39%, respectively), TALA was generally well-tolerated and non-hematologic adverse events were less common [114]. Additionally, participants in the EMBRACA phase 3 trial showed significant delay in time to definitive clinically-meaningful deterioration (TTD) compared to patients treated with physician’s choice of chemotherapy (PCT), according to results using the European Organization for Research and Treatment of Cancer Quality of Life Questionnaire Core 30 (EORTC QLQ-30) [115]. Based on the results of EMBRACA, on October 16, 2018, the U.S. FDA granted approval of talazoparib for treatment of patients with BRCA-mutated, HER2-negative, advanced or metastatic breast cancer.

## 8. Acquired Resistance to PARP Inhibitors

Although PARP inhibition is a promising therapeutic approach for *BRCA*-mutated breast cancers, in some cases PARPi resistance can emerge, often via poorly understood mechanisms. One such mechanism for PARPi resistance involves upregulation of Pgp, a drug efflux transporter [116]. AZD2461 is a next-generation PARP inhibitor that was synthesized as a poor substrate for Pgp. This compound was developed in response to olaparib resistance and was derived from the same chemical series as olaparib, the piperidine ether series [117]. 

Inactivation of p53-binding protein 1 (53BP1) has also been implicated in the development of resistance to DNA-damaging agents by partially restoring HRR and rescuing proliferation defects in *BRCA1*-deficient cells [118]. Data shows that reduced 53BP1 expression is correlated with TNBC and in *BRCA1/2* mutant tumors [118]. Expanding upon the role of 53BP1 in promoting PARPi cytotoxicity, 53BP1 inhibits HRR by impairing 5’ end resection [118,119]. More recent research points to the interaction of 53BP1 with Rif1 to block resection of DNA breaks [120]. In a dominant negative 53BP1 construct, it was shown that 53BP1 suppression decreased NHEJ and increased HRR, which suggests that 53BP1 may regulate the choice between the two repair pathways [121]. In an in vivo model, Jaspers et al. report resistance to PARPi due to somatic loss of 53BP1 and partial restoration of HRR [116]. Another in vivo model from Li et al. examines mutant BRCA1 lacking the N-terminal RING domain. In cells expressing RING-less BRCA1, RAD51 foci still form in response to DNA damage. In mice which carry exon 2-deleted forms of *BRCA1*, Li et al. found that ablation of 53BP1 rescues embryonic lethality, although other *BRCA1* phenotypes were not rescued by 53BP1 deletion. Their findings provide support that 53BP1 and BRCA1 collaborate to maintain genomic integrity [122]. Further implications toward resistance mechanisms can be drawn from these studies, as cells which lack 53BP1 and deletion of N-terminal-deleted BRCA1 isoforms can contribute to chemoresistance. 

Given that RAD51 is an important component of the HRR pathway, elevated levels of RAD51 have been shown to contribute to PARPi resistance [123]. In this line of thinking, factors that increase RAD51 levels may also contribute to resistance. Marzio et al. have recently demonstrated that early mitotic inhibitor 1 (EMI1) activity targets RAD51 for degradation [124]. EMI1 is a substrate of S phase kinase-associated protein 1 – cullin 1 – F-box protein (SCF) ubiquitin ligase, and functions as an APC/C (anaphase-promoting complex or cyclosome) inhibitor [125,126]. In this 2019 study, Marzio et al. found that EMI1 assembles a SCF ubiquitin ligase complex to target RAD51. They showed that a subset of TNBC cells downregulate EMI1, which restores RAD51-dependent HRR, and these cells develop resistance to PARPi. Additionally, they reconstituted EMI1 in cells and in a mouse model and found that PARPi sensitivity was reestablished [124].

Genetic reversion of *BRCA1* or *BRCA2* mutations is another proposed mechanism of PARPi resistance. In patients that had previously been treated with olaparib, and received initial response followed by progressive tumor growth and a marked increase in CA15-3 (a carcinoma antigen), a secondary *BRCA2* c.9106C>G was detected. In a germline *BRCA2* c.9106C>T mutation, a stop codon is introduced. With this secondary point mutation, the *BRCA2* open reading frame is potentially restored, and the functional BRCA protein restores HRR [127]. Because the sample size in this study was small, more preclinical data is needed to determine whether secondary mutations in *BRCA* are dominant mechanisms for resistance to PARP inhibition. 

Another theory of resistance to PARPi involves the upregulation of enhancer of zeste homolog 2 (EZH2), which is the catalytic component of the polycomb repressive complex 2 (PRC2) [128]. PRC2 and EZH2 are involved in histone methyltransferase activity and transcriptional repression. PARP and EZH2 directly interact, and their interaction is upregulated by PARP-1 activation and inhibited by PARPi. Results show that EZH2 and H3-K27me3 have increased expression in resistant cells. Thus, a combination of PARPi and EZH2i has been shown to decrease the cancer stem cell population and colony formation in vitro and tumor size in vivo [128]. Interestingly, ER+ breast cancer patients with low H3K27me3 and high EZH2 have shorter overall survival and poor prognosis [129].

A very recent investigation has identified point mutations in PARP-1, which confer PARPi resistance [130]. The researchers first confirmed that ablation of PARP-1 expression did, in fact, hinder trapping of PARP-1 and confers PARPi resistance. The resistance was due to in-frame PARP-1 mutations in *Parp1,* which disrupt the interaction of zinc finger (ZnF) domains at the site of DNA and PARP-1. Using a CRISPR-Cas9 screen, they identified candidate PARP-1 amino acid residues associated with resistance, K119 and S120, which are involved in the binding of DNA residues to the second ZnF domain of PARP-1. Mutations were also identified outside of the ZnF domain, such as those in amino acid residues in the DNA-binding and catalytic domains [130]. Additionally, it has been shown that PARP catalytic activity is increased upon phosphorylation of PARP-1 on amino acid Tyr907. This in turn reduces PARPi binding [131].

Several research groups point to increased replication fork stability as a contributor to PARPi resistance in BRCA1/2-deficient cancers. In the presence of a DNA strand break and the absence of PARP-mediated DNA repair, replication forks collapse into a DSB [29]. However, when the replication fork is stabilized and alternate mechanisms of repair dominate, resistance to PARPi occurs. Increased replication fork stability may occur through loss of PTIP (Pax2 transactivation domain-interacting protein, an HRR protein) [132], restoration of HR [133], or through the EZH2/MUS81 axis [134]. The EZH2/MUS81 axis characterizes the recruitment of the MUS81 nuclease when EZH2 localizes at stalled replication forks and increases methylation of H3K27. When EZH2 levels are low; however, methylation is reduced and replication forks are stabilized as MUS81 recruitment is prevented [134]. 

Additional proposed mechanisms of resistance include increased expression of miR-493-5p, which induces resistance to platinum and PARPi [135], and also depletion of PARG, the major enzyme involved in the catabolism of PARP [136]. Given many recent discoveries in the field of PARPi resistance, further investigation is warranted to improve treatment strategies for cancer patients. The existence of multiple mechanisms of resistance to PARPi highlights the need for functional biomarkers and strategies involving combination treatment.

## 9. Conclusions

Because breast cancer is a widely heterogeneous disease, treatment is complicated and often involves multiple therapeutic modalities, such as chemotherapy, surgery, and radiation. Although PARP inhibition is a promising therapeutic strategy to treat breast cancer, PARP inhibitors have clinical activity restricted to a small category of patients with *BRCA* mutations. The challenge is to determine the differences in breast cancer cell line responsiveness to PARP inhibition. To develop personalized therapeutic targets, detailed characterization of breast cancer cells is required, along with the use of predictive biomarkers and diagnostic methods mentioned in this review. To complicate matters further, present studies mentioned in this review suggest that PARPi sensitivity is not restricted to breast cancer with BRCA mutations. With two PARPi approved by the FDA to treat breast cancer, the future is promising for the development of other PARP inhibitors for targeted therapy. Clinical studies are underway examining PARPi as neoadjuvant therapy, as monotherapy, and in combination therapy.

## Abbreviations

aNHEJ	alternative non-homologous end-joining
BER	base excision repair
BID	bis in die, twice a day
*BRCA1/2*	breast cancer type 1/type 2 susceptibility protein
CNA	copy number alterations
CAT	catalytic domain (of PARP-1)
CBR	clinical benefit rate
CGH	comparative genomic hybridization
CR	complete response
DLT	dose-limiting toxicities
DNMTi	DNA methyltransferase inhibitor
DSB	double-strand break
EMI1	early mitotic inhibitor 1
ER	estrogen receptor
EZH2	enhancer of zeste homolog 2
gBRCAm	germline BRCA mutation-associated
HD	helical domain
HER2	human epidermal growth factor receptor 2
HRD	homologous recombination deficiency/homologous recombination-deficient
HR	hormone receptor
HRR	homologous recombination repair
IRIF	ionizing radiation induced foci
IV	intravenous
LOH	loss of heterozygosity
LST	large-scale state transitions
miRNA	micro RNA
NAD^+^	nicotinamide adenine dinucleotide
NHEJ	non-homologous end-joining
ORR	objective response rate
PARP	poly (ADP-ribose) polymerase
PARPi	poly (ADP-ribose) polymerase inhibitor
pCR	pathological complete response
PCT	physician’s choice of chemotherapy
PFS	progression-free survival
PO	per os, by mouth
PR	progesterone receptor
PRC2	polycomb repressive complex 2
PRR	partial response rate
QD	quaque die, once a day
RER	ribonucleotide excision repair
ROS	reactive oxygen species
RP2D	recommended phase 2 dose
SD	stable disease
SNP	single-nucleotide polymorphism
SSA	single-strand annealing
TAI	telomeric allelic imbalance
TNBC	triple negative breast cancer
TOP1	topoisomerase 1
